# Relationship of left atrial mechanics to electrical activity on surface electrocardiography in idiopathic dilated cardiomyopathy

**DOI:** 10.21542/gcsp.2019.7

**Published:** 2019-03-31

**Authors:** Hala Mahfouz Badran, Naglaa Faheem, Kerolos Wagdy Wassely, Magdi Yacoub

**Affiliations:** 1 Cardiology Department Menoufiya University, Egypt; 2 The BAHCM National Program, Egypt; 3 Aswan Heart Center, Aswan, Egypt; 4 Imperial College, London, UK

## Abstract

**Aim:** (1) Assess left atrial (LA) mechanics and electromechanical delay in patients with idiopathic dilated cardiomyopathy (IDCM), and (2) examine the relationship between atrial electromechanical delay and atrial electrical activity [P-wave duration, P-wave dispersion (PWD) and P terminal force (PTF)] on surface ECG.

**Methods:** 73 IDCM patients (age 36  ±  17 years); 63% men,25 age & sex matched healthy subjects were studied. LA atrial electromechanical delay & mechanics (ε_sys_, SR_sys_, SR_e_, SR_a_) were measured with 2D-strain. From 12-lead electrocardiograms, P-wave duration, PWD and PTF calculated.

**Results:** Reservoir, conduit and contractile functions were predominantly reduced compared to control (*P* < 0.001). Intra-atrial electromechanical delay was 88.9 ± 84.6 in IDCM versus 27.4 ± 16.5 in control (*P* < 0.0001). In IDCM, PWD (52.89 ± 15), P_max_(98 ± 17.5) and PTF(58.2 ± 36) were significantly increased compared to control (36.20 ± 8.9, 79 ± 9.9, 25.22 ± 8.76) respectively (*P* < 0.0001). A positive correlation was detected between intra-atrial electromechanical delay and PWD &PTF (*r* = 0.5, *P* < 0.0001). By stepwise multiple linear regression analyses, LA reservoir function (LA ε_sys_) [β = 0.754; CI at 95%:0.356–0.780, *P* < 0.001] and LA volume [β = 0.743; CI 95%:0.423–0.75, *P* < 0.001], and PWD [β=0.848; CI 95%:0.311–0.644, *P* < 0.0001], and PTF [β = 0.927; CI 95%: 0.357–0.722, *P* < 0.0001] are independent predictors for LA electromechanical delay in IDCM.

**Conclusion:** In addition to altered LA mechanics, atrial electromechanical delay gets longer in IDCM and is correlated with PWD and PTF. Atrial electrical dispersion on surface ECG could be early index of LA dysfunction that deserves further study.

## Introduction

P-wave dispersion (PWD), defined as the difference between the maximum and minimum P-wave duration on surface ECG, is a new electrocardiographic marker that has been associated with inhomogeneous and discontinuous propagation of sinus impulses^1^. The correlation between the presence of inter-atrial and intra-atrial conduction abnormalities and the induction of paroxysmal atrial fibrillation (AF) has been well documented^2^.

In addition, prolonged P-wave duration and increased PWD are commonly found in patients with a history of paroxysmal AF^3^. AF, whether chronic or paroxysmal, is the most common sustained arrhythmia encountered in clinical practice that produces substantial excess cardiovascular morbidity and mortality^3^. The estimation of the probability of a patient in developing AF paroxysms, might guide the clinician in the management and stratification of patients at higher risk of developing AF.

In dilated cardiomyopathy, atrial dilatation and fibrosis are characteristic findings that cause atrial conduction abnormalities^4,5^.

Recently, strain and strain rate – measured by novel speckle-tracking echocardiography (2D-STE) – have been used in evaluating cardiac mechanics. It allows simultaneous and precise analysis of atrial mechanics during different phases of reservoir, conduit and contractile functions^5,6^ in addition to measurement of atrial EMD, from the onset of the P-wave on ECG, to the onset of atrial contraction.

The objectives of our study were to investigate the relation of atrial electrical activity using P-wave dispersion and P terminal force on surface ECG to LA electromechanical delay and mechanics using 2D strain imaging in idiopathic dilated cardiomyopathy (IDCM).

## Patients and Methods

### Study population

#### IDCM group

Between January 2013 and December 2015, we prospectively recruited 73 patients with IDCM, who were referred to our echocardiographic laboratory in a single center (Yacoub Research Unit, Menoufia University, Egypt). Patients were enrolled in the study after their informed consent, and approval of Ethics Committee of Menoufia University Hospitals was obtained.

IDCM was defined by the presence of LV dilatation and systolic dysfunction in the absence of abnormal loading conditions such as valve disease, coronary artery disease sufficient to cause global systolic impairment^7^.

The diagnosis of IDCM was based on conventional echocardiographic features, including ejection fraction <45%, and/or fractional shortening <25%, in association with LV end-diastolic dimension >112% of predicted value corrected for age and body surface area^7^.

Patients mean age was 36 ± 17, all have sinus rhythm. Exclusion criteria were: ischemic heart disease (using coronary angiography in patients >40 years), congenital heart diseases, primary valvular heart diseases, other causes of cardiomyopathy (hypertrophic/restrictive), atrial fibrillation, lung or metabolic disorders, permanent pacemaker or cardiac resynchronization therapy (CRT) and inadequate echocardiographic studies.

The medical treatment of IDCM patients included *β*-blockers (*n* = 30, 41%), angiotensin-converting enzyme inhibitors (*n* = 67, 94.5%), spironolactone (*n* = 52, 71%), angiotensin receptor blockers (*n* = 3, 4%), diuretics (*n* = 70, 96%), digoxin (*n* = 22, 30%), and nitrates (*n* = 10, 13.7%).

#### Control group

We studied 25 age and sex-matched healthy subjects, without detectable cardiovascular risk factor or receiving any medication with a normal 12-lead ECG and echocardiography. Volunteer controls were all selected from departments of adult cardiology and among subjects investigated for work eligibility.

The study population was subjected to complete clinical evaluation, exclusion of other causes of cardiomyopathy and assessment of NYHA functional class.

### ECG analysis

ECG analysis was performed using 12-lead ECG and 12SL ECG analysis program from ESAOTE Organizer. All resting ECGs were recorded at 50 mm/s and 20-mm/mV standardization with standard lead positions. One cardiologist, who was blinded to the clinical status of the patients, measured the ECG intervals for each study patient.

Measurements included the atrial electrical activity, RR and QRS intervals, they were confirmed manually and determined in all limb and precordial leads. The QRS duration was measured from the beginning of QRS complex to its end.

Atrial electrical activity included: P-wave amplitude, PR interval, P-wave dispersion (PWD). All derivations were measured. The onset of the P-wave was defined as the point of the first visible upward slope from baseline for positive waveforms and first downward slope from baseline for negative waveforms. The return of P-wave deflection (where it intersects the isoelectric line) was considered to be the end of the P-wave. *P*_max_ was the longest and *P*_min_ was the shortest atrial conduction time measured in any of 12 leads ECG and the difference between them was PWD. The derivations were excluded if the beginning or ending of the P-wave could not be clearly identified^7,8^.

Intra-observer and inter-observer coefficients of variation were found to be 4.1% and 4.4% for PWD, respectively^7,8^. P-wave terminal force (PTF) is the duration in millisecond of the terminal part (negative) of the P-wave in lead V1 multiplied by its depth (in millimeters)^10–12^.

### Conventional echocardiographic study

Echocardiographic examinations were performed in the left lateral decubitus, in the parasternal long, short-axis, apical two- and four-chamber views using standard transducer positions. Esaote Mylab Gold 30 ultrasound system (Esaote S.p.A, Florence, Italy) equipped with a multi-frequency 2.5–3.5 MHz phased-array transducer was utilized. Cardiac dimensions, volumes and functions were measured in accordance with the recommendations of the American Society of Echocardiography^9^. Left ventricular end diastolic (EDD), end systolic diameter (ESD), septum (ST) and posterior wall thickness (PWT) were measured. Ejection fraction (EF%) was calculated using the Simpson biplane method. Transmitral flow velocities (E and A) were obtained by pulsed-wave Doppler at mitral leaflets tip in the apical four-chamber view. The ratio of E/A velocity and E-wave deceleration time (DT) were measured.

Color flow mapping and continuous wave Doppler were used to estimate pulmonary artery pressure (PAP) from tricuspid regurgitation velocity Systolic PAP was calculated from the tricuspid regurgitant jet velocity (V) using the Bernoulli equation (4V^2^) and the estimated right atrial pressure^9^.

Tissue Doppler imaging was used to measure mitral annular velocities. The peak systolic (S’), early diastolic (E’), atrial diastolic (A’) velocities were measured at both the mitral septal and lateral annulus, and the mean was taken. The ratio of E/E’ was calculated by using average E’.

The LA diameter was measured by M-mode in parasternal long-axis view at end-systole, at the maximum dimension from the leading edge of the posterior wall of the aorta to the dominant line of posterior wall of LA. LA volume was measured at LV end systole, from the apical four-chamber and two chamber views according to the biplane area-length method as described previously^10^.

### Analysis of LA mechanics using 2D strain imaging

LA images were recorded and processed. Tracking and subsequent strain calculations were performed with the software package Esaote-X-Strain™ based on a previously validated algorithm^10^. Scanning was performed longitudinally from the apex to acquire best apical views. Frame rate (70 ± 20 F/s), was adjusted depending on the heart rate.

Border tracking of the LA was manually traced from the digitized 2D video clips recorded during breath holding and with good quality ECG signals, which were acquired and stored for off-line analysis. The “Zoom/RES” feature on the echocardiographic machine was used to improve the accuracy of atrial measurements. A circular region of interest was traced on the endocardial cavity interface of the apical four-chamber view at end diastole (LA minimum cavity area) using a point-and-click approach. Time-volume curves were extracted from LA wall tracking that provided automatically indexed maximum and minimum LA volume and left atrium ejection force (LAEF). We measured longitudinal peak velocities achieved by LA walls 1 cm above the mitral annulus in systole (Sam), early (Eam) and late diastole (Aam). Definition of the LA endocardial border enabled the system to calculate regional longitudinal deformation of the LA walls^11^.

LA peak systolic strain (ε_sys_) and LA systolic SR (SR_sys_) were measured as a positive curve at LV systole (representing reservoir function), early diastole (SR_e_) (representing conduit function), and atrial diastole (SR_a_) (representing contractile function). Image processing algorithms automatically subdivide the atrial wall into segments distributed in septum and lateral, and posterior LA wall, or “roof”. The graphs for each segment were displayed and averaged to calculate global LA function (Figure 1A).

**Figure 1A.**
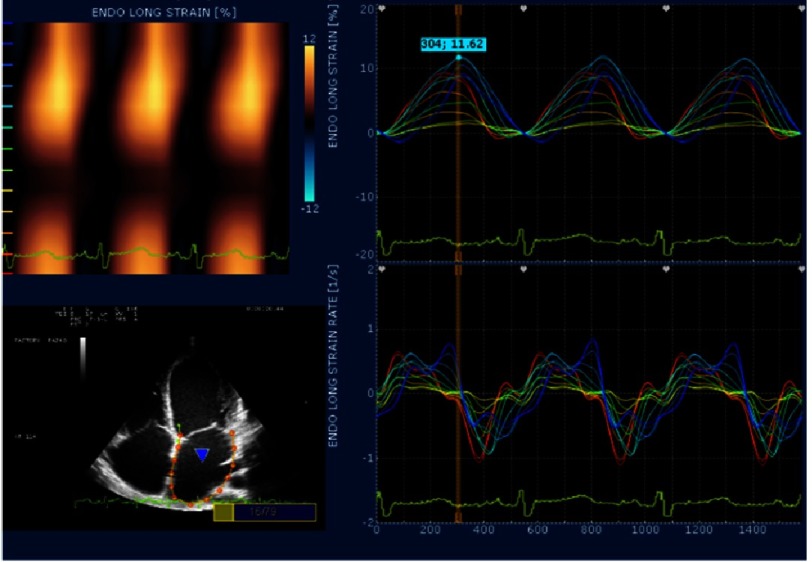
Measurements of LA ε_sys_ andSR obtained from four-chamber using VVI. Peak positive LA ε_sys_, and peak positive SR_sys_ corresponds to reservoir function, LA peak positive early diastolic SR(SR_e_) corresponds to conduit function, peak positive atrial diastolic SR (SR_a_)corresponds to LA contractile function.

**Figure 1B.**
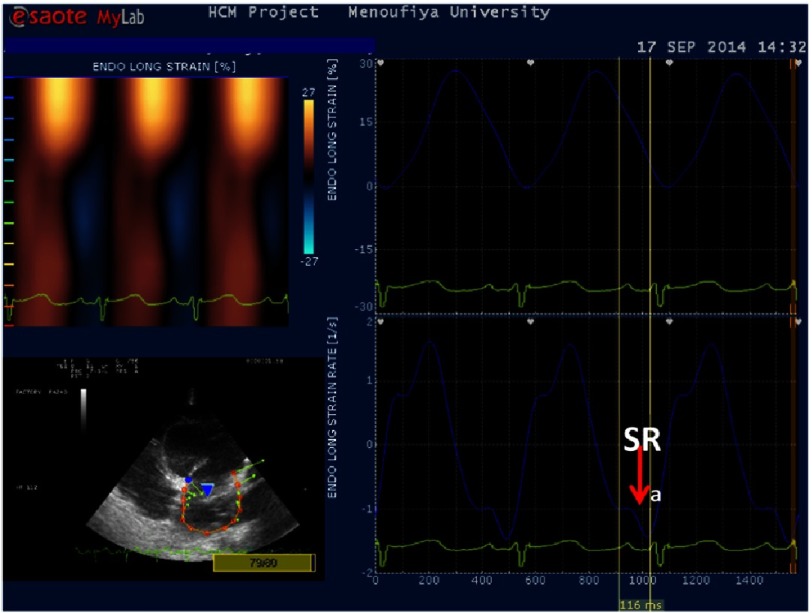
LA Strain and (top right) strain rate (bottom right) curve showing measurement of TTP from the beginning of P-wave on ECG to peak SR_**a**_ in IDCM (TTP=116 ms).

To estimate LA mechanical dyssynchrony, the index of myocardial activation was calculated from regional strain rate curves for each segment, as time from the beginning of P-wave of ECG to the peak longitudinal atrial diastolic SR (TTP) (Figure 1B). Left atrial electromechanical delay (d-TTP) was measured as the difference between TTP in 12 LA segments (difference between the longest and shortest cycle). Intra-atrial dyssynchrony was defined as the standard deviation of the averaged time-to-peak SR_a_ (TTP-SD)^12^.

### Statistical analysis

Data were presented as numbers (%) or mean ± SD, as indicated. The distribution of qualitative variables was evaluated by Chi Square test or Fisher’s exact test, as indicated. Differences between means were compared by Students’ test or one-way Analysis of variance, as appropriate. Correlation between numerical variables was assessed by Pearson’s correlation coefficient, r. Statistical analysis was done by IBM SPSS statistical software package for Mac, version 22. All tests were bilateral and the limit of statistical significance was 0.05.

## Results

### Clinical characteristics of the study population

The clinical characteristics of the 73 IDCM patients [36 ± 17y, 46 (63%) were men] and controls are shown in Table 1.

**Table 1:** Conventional echocardiographic characteristics.

Echocardiographic characteristics:	DCM (*n* = 73)	Control (*n* = 25)
Age	36.2 ± 17.2	34.2 ± 17
Male	46 (63%)	14(56%)
Female	27(37%)	11(44%)
BSA (kg/m^2^)	1.68 ± 0.48	1.51 ± 0.51
Familial DCM	14 (19.2%)	
Functional class		
NYHA I	3 (4.1%)	
NYHA II	32 (43.8%)	
NYHA III	30 (41.1%)	
NYHA IV	8 (11%)	
Duration of symptoms (months)	22 ± 37	
NYHA class	2.56 ± 0.77	
Heart rate (b/min)	87.3 ± 15.6	78.4 ± 12.3^a^
SBP (mmHg)	116.4 ± 17.3	119.4 ± 8.2
DBP (mmHg)	73.9 ± 11.9	76.2 ± 8.5
LA diameter(mm)	40.8 ± 12.10	27.2 ± 6.17^a^
LAV (ml)	61.55 ± 31.70	23.44 ± 11.5^a^
LAV index (ml/m^2^)	33 ± 15.45	15.50 ± 5.45^a^
ESD (mm)	56.34 ± 12.44	28.94 ± 5.40^a^
EDD (mm)	66.77 ± 13.17	45.59 ± 8.21^a^
FS %	16.1 ± 5.8	34.76 ± 8.78^a^
EF %	32.9 ± 10.1	65.76 ± 6.53^a^
Septum (mm)	9.03 ± 2.18	8.54 ± 1.69
LVPW (mm)	8.74 ± 2.8	8.99 ± 1.72
LVM (gm)	328.5 ± 166.3	163.3 ± 87.3^a^
LVM index (g/m^2^)	194.5 ± 77.8	102.3 ± 31.7^a^
E Mitral(cm/sec)	86.8 ± 35.1	80.73 ± 14.9
A Mitral (cm/sec)	52.6 ± 21.8	52 ± 21
E/A mitral	1.9 ± 1.3	1.5 ± 0.3^b^
DT (ms)	149.7 ± 55	170.8 ± 32
E’(cm/s)	8.4 ± 3.6	17 ± 3.2^a^
E/E’	11.8 ± 5.5	4.8 ± 0.6
PAP( mmHg)	33.2 ± 11.8	18.4 ± 2.8^a^
Mitral Regurg:		
No	0	15 (60%)
Trivial	11 (15%)	10 (40%)
Mild	27 (37%)	0
Moderate	21 (28.7%)	0
Severe	14 (19.3)	0
Tricuspid Regurg:		
No	3 (4.1%)	19 (76%)
Trivial	25 (34.2%)	6 (24%)
Mild	30 (41.1%)	0
Moderate	10 (13.7%)	0
Severe	4 (5.4%)	0

**Notes.**

a *P* < .001.

b *P* < .01.

LAD left atrium diameter LAV left atrial volume PWT posterior wall thickness EDD left ventricular end diastolic diameter ESD left ventricular end systolic diameter FS fractional shortening EF% ejection fraction LVMI left ventricular mass index E early diastolic mitral inflow velocity A atrial diastolic mitral inflow velocity DT deceleration time E’ early diastolic myocardial velocity PAP pulmonary artery pressure

**Table 2:** Left atrial mechanics in the studied groups.

	DCM (*n* = 73)	Control (*n* = 2)
ε_sys_ Global (%)	12.8 ± 11.4	39.6 ± 19.4^a^
SRsys Global s^−1^	0.76 ± 0.45	1.84 ± 0.55^a^
SRe Global s^−1^	−0.63 ± 0.64	−2.04 ± 0.69^a^
SRa global s^−1^	−0.71 ± 0.45	−1.23 ± 0.60^a^
TTP- d (ms)	88.9 ± 84.6	27.4 ± 16.5^a^
LA TTP-SD	43.7 ± 38.1	13.9 ± 10.4^a^
LA volume max (ml)	109.24 ± 55.9	52.03 ± 25.93^a^
LA volume min (ml)	78.03 ± 48.04	20.67 ± 15.32^a^
LA EF %	32.29 ± 16.97	62.04 ± 12.15^a^

**Notes.**

a *P* < .001.

ε_sys_ Peak systolic strain SR_sys_ Peak systolic strain rate SR_e_ early diastolic strain rate SR_a_ atrial diastolic strain rate TTP-d difference of time to peak atrial SR TPP-SD standard deviation of time to peak atrial SR LA left atrium

59 (80.8%) patients were ≤50 years age. The disease was familial (at least one affected first-degree relative) in 14 patients (19.2%). 6 patients had a family history of sudden cardiac death. Regarding the symptoms at presentation, only 3 (4.1%) patients had good functional status (NYHA class I), 32 (43.8%) had mild symptoms (class II), 30 (41.1%) were moderately symptomatic (class III), and 8 patients (11%) had severe symptoms even at rest (class 4). The mean duration of symptoms was 22 months.

On conventional echocardiography, LV end diastolic and end systolic diameters were significantly higher in IDCM patients while LV EF% was considerably lower in comparison to control (*P* < 0.001). LV end diastolic pressures as estimated by E/E’ showed marked deterioration in IDCM group (11.8 ± 5.5 versus 4.8 ± 0.6, *P* < 0.001) compared with control group.

There were 14 (19%) IDCM patients have severe mitral regurgitation, 21(28.7%) had moderate MR, while 38 (52%) had insignificant mitral regurgitation. Moreover, 4 (5%) IDCM patients had severe and 10 (14%) had moderate tricuspid regurgitation.

Concerning LA assessment by conventional echocardiography, IDCM patients showed significant increase in anterio-posterior diameter and LAV (40.8 ± 12.1 vs 27.2 ± 6.2 cm) and (61.55 ± 31.7 vs 23.4 ± 11.5 ml) compared to control (*P* < 0.001). 39 (53%) IDCM patients had LA diameter >4 cm, 13 (18%) had LA diameter >5 cm and 44 (60%) had LAV index>34 ml/m^2^.

### LA mechanical functions

The comparison of LA strain indices among the study groups is shown in Table 2. The reservoir function, as represented by LA ε_sys_ and LA SR_sys_ was significantly reduced in IDCM patients (12.82 ± 11.43%, 0.76 ± 0.45 s ^−1^) compared with control group (39.63 ± 19.39 %, 1.84 ± 0.55 s ^−1^) (*P* < 0.001). Similar results were observed for LA conduit function as assessed by LA SR_e_ and contractile function, as reflected by SR_a_ (*P* < 0.001). From LA longitudinal strain curves, LA EF% was derived as the difference between LA maximum and minimum volume. It was significantly reduced in IDCM compared to control group (32.29 ± 16.97 versus 62.04 ± 12.15), *P* < 0.001.

### Atrial electromechanical delay using 2D strain

An inverse trend was found for intra-A dyssynchrony. LA electromechanical delay (d-TTP) measured as the difference between longest and shortest TTP was significantly increased compared to control group (*P* < 0.001). Intra-A dyssynchrony, as expressed by TTP-SD, was predominantly increased in IDCM (43.69 ± 38.1) in comparison to control group (13.99 ± 10.39), *P* < 0.001.

### Relation of LA mechanical dispersion to LA functions

Taking IDCM patients altogether, electromechnical delay (TPP-d) was negatively correlated with both reservoir function, LA ε_sys_ (*r* = 0.28, *P* < 0.02) and conduit function (SR_e_: *r* = 0.28, *P* < 0.02). In addition LA mechanical dyssynchrony (TTP-SD) was inversely related to LA ε_sys_ & SR_sys_ (*r* =  − 0.31, *P* < 0.01) and LA SR_e_ (*r* =  − 0.38, *P* < 0.003) and also correlated to contractile function (SR_a_) (*r* =  − 0.27, *P* < 0.02).

**Table 3:** Electrocardiographic characteristics in studied groups.

ECG data	IDCM (*n* = 73)	Control (*n* = 25)
P amplitude mv	1.84 ± 0.52	1.32 ± 0.20^a^
*P*_max_ (ms)	98.03 ± 17.49	79 ± 9.895^a^
*P*_min_ (ms)	45.27 ± 10.24	42.8 ± 7.23
PW dispersion (ms)	52.89 ± 15	36.20 ± 8.9^a^
PR interval (ms)	167.34 ± 30.21	133.68 ± 14.89^a^
PTF (ms.mv)	58.20 ± 35.97	25.22 ± 8.76^a^
LBBB	15(21%)	0
Incomplete LBBB	6(8.2%)	0
RBBB	1(1.4%)	0
IVCD	6(8.2%)	0
No	48(65.8%)	25(100%)
Voltage criteria		
Yes	28(38.4%)	0
No	45(61.6%)	25(100%)

**Notes.**

a *P* < .0001.

*P*_max_ longest P-wave *P*_min_ shortest P-wave PW P-wave dispersion PTF P terminal force measured in lead V1 LBBB left bundle branch block RBBB right bundle branch block IVCD intra-ventricular conduction delay

### Electrocardiographic measurements in studied subjects

**Figure 2.**
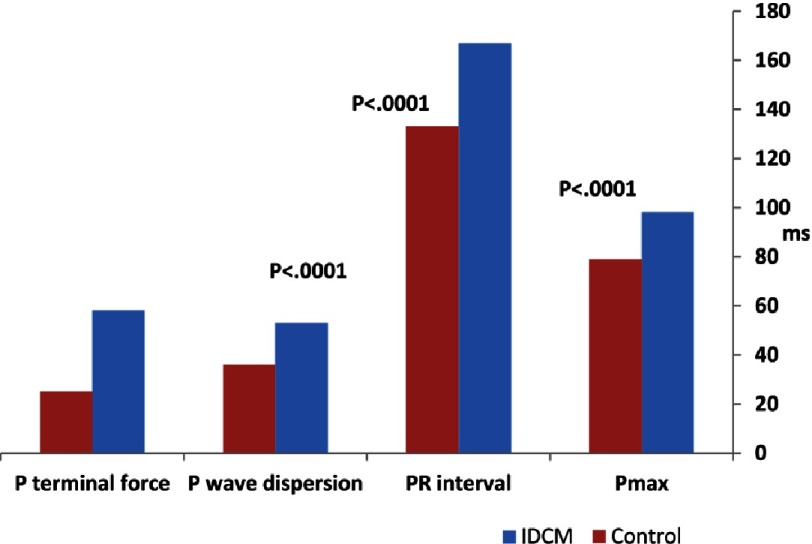
Comparison of atrial electrical activity in IDCM & control.

The P-wave amplitude, PR interval, *P*_max_ and PWD were significantly prolonged in IDCM compared with control (*P* < 0.001). Additionally, in IDCM P-wave terminal force (58.20 ± 35.97) were significantly increased in comparison to control (25.22 ± 8.76), *P* < 0.001 respectively (Table 3, Figure 2).

### Relation of LA echocardiographic parameters to electrocardiographic variable

In the control group, univariate analysis showed a modest positive correlation between PTF and LAV (*r* = 0.39, *P* < 0.04), E/E’ (*r* = 0.42 *P* < 0.03) and LA electromechanical delay (r=0.49, P<0.01). However, there was no significant correlation between PWD and any of echocardiographic variables.

On the contrary, in IDCM patients, PTF showed strong relation with most LA conventional and deformation parameters. PTF had strong positive relationship with age (*P* < 0.005), LA volume index (*P* < 0.001), and negative correlation to LA EF (*P* < 0.003). PTF showed modest correlation with ESD (*P* < 0.01), EDD (*P* < 0.03) but strong negative correlation with LV EF% (*P* < 0.006). Additionally, general linear modeling analysis revealed significant interactions between PTF and LA mechanics*.*

PTF was negatively correlated to LA reservoir function (ε_sys_: *P* < 0.005, SR_sys_: *P* < 0.01) and LA conduit function as reflected by SR_e_ (*P* < 0.003). Also there was strong positive correlation between PTF and LA electromechanical delay (TTP-d) (*r* = 50, *P* < 0.0001). However, the relation between PTF and LA contractile function as measured by (SR_a_) did not exist. On the other side, no significant correlation was detected between PTF and LA dyssynchrony (TTP-SD).

Considering PWD, it showed a strong direct correlation with age (*P* < 0.0001), EDD & ESD (*P* < 0.0001), LA volume (*P* < 0.0001), and LA TTP-d (*P* < .00001). However, it did not show other significant relationship with the LA mechanics or LV function.

*P*_max_ (representing the P-wave duration) showed direct correlation with age, LA diameter, LA volume, ESD, EDD (*P* < 0.0001) and modest inverse correlation with atrial conduit function (SR_e_) (*P* < 0.03).

The PR interval showed direct correlation to age, LA diameter, LA volume, ESD, EDD (*P* < 0.001). Conversely, it showed inverse correlation with LA mechanics: ε_sys_ (*P* < 0.01), SR_sys_ (*P* < 0.01), SR_e_ (*P* < 0.004), and SR_a_ (*P* < 0.02). Results are summarized in Table 4 and Figures 3A–3G.

**Table 4:** Relation of atrial electrical activity to echocardiographic variables.

	PTF		PWD		*P* max		PR interval	
	*r*	*P* value	*r*	*P* value	*r*	*P* value	*r*	*P* value
Age	.327	.005	.456	.000	.445	000	.227	.021
NYHA class	−.110	.361	.136	.259	.133	.267	.327	.005
LA EF%	−.339	.003	−.160	.178	−.187	.112	−.234	.047
LA diameter	.376	.001	.258	.028	.367	.001	.360	.002
LA volume(ml)	−.356	.005	.441	.000	.533	000	.465	.000
LA volume (ml/m^2^)	−.384	.001	.440	.000	.243	.021	.387	.001
ESD (cm)	.300	.010	.438	.000	.440	000	.328	.005
EDD (cm)	.248	.034	.495	.000	.466	000	.333	.004
LV FS%	−.317	.006	−.069	.559	−.132	.266	−.169	.152
LV EF%	−.318	.006	−.125	.292	−.150	.204	−.142	.231
E/E’	−.066	.579	−.014	.908	.029	.805	−.032	.789
DT (ms)	−.006	.962	−.016	.893	.023	.849	−.054	.650
PASP (mmHg)	.004	.974	.171	.149	.117	324	.047	.671
ε_sys_ LA Global	−.323	.005	−.173	.144	−.199	.091	−.282	.012
SR_sys_ LA Global	−.276	.018	−.097	.416	−.144	.224	−.282	.015
SR_e_ LA Global	−.346	.003	.209	.074	−.247	.035	.336	.004
SR_a_ LA Global	.121	.309	.062	.601	.120	.310	.265	.024
LA TPP-SD	.091	.443	.160	.177	.199	.091	.023	.847
LA TPP-d	.501	.0001	.452	.000	.181	.126	.019	.875

**Notes.**

LAD left atrium diameter LAV left atrial volume EDD left ventricular end diastolic diameter ESD left ventricular end systolic diameter FS fractional shortening EF% ejection fraction E/E’ early diastolic mitral inflow velocity to early diastolic myocardial velocity DT deceleration time PAP pulmonary artery pressure ε_sys_ Peak systolic strain SR_sys_ Peak systolic strain rate SR_e_ early diastolic strain rate SR_a_ atrial diastolic strain rate TTP-d difference of time to peak atrial SR TPP-SD standard deviation of time to peak atrial SR

**Figure 3A.**
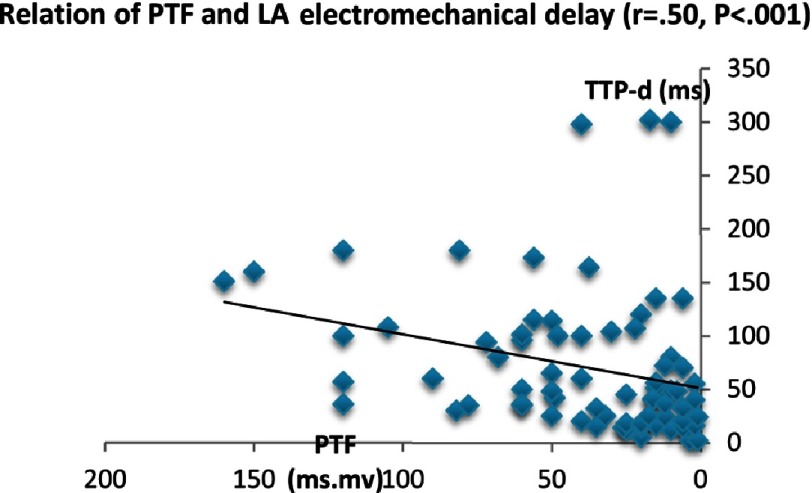
Relation of PTF and LA electromechanical delay.

**Figure 3B.**
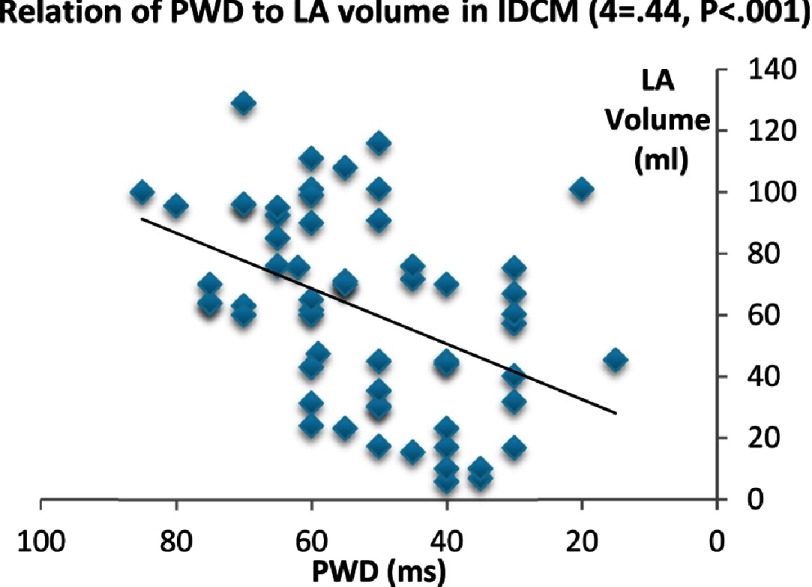
Relation of PWD to LA volume in IDCM.

**Figure 3C.**
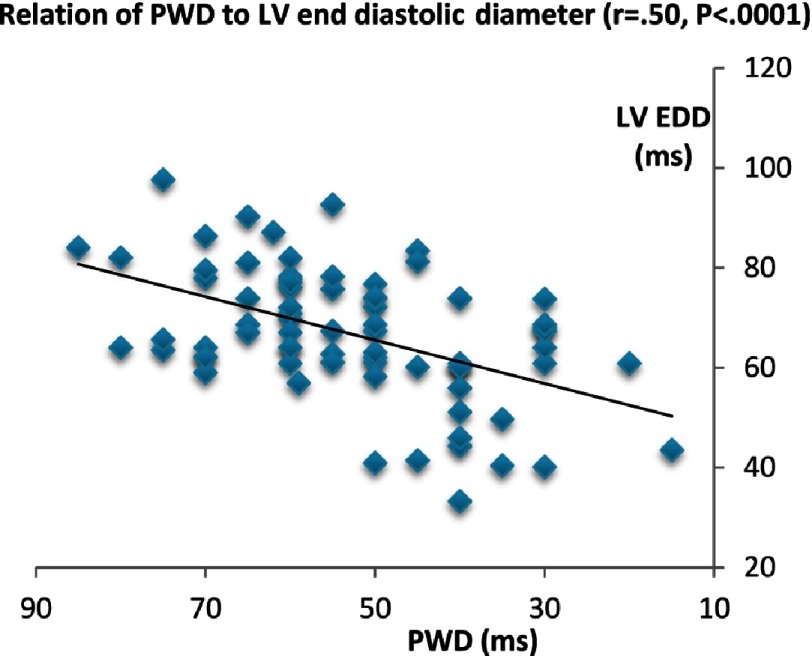
Relation of PWD to LV end diastolic diameter.

**Figure 3D.**
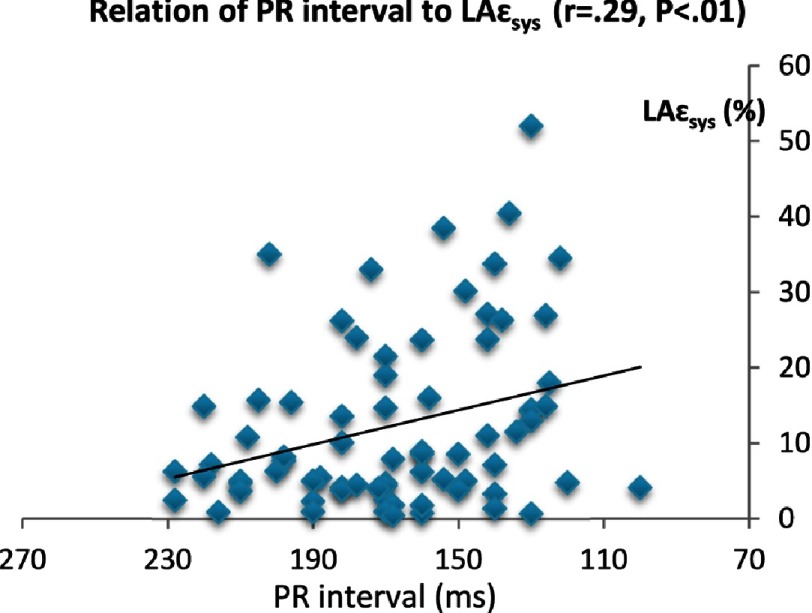
Relation of PR interval to LAε_**sys**_.

**Figure 3E.**
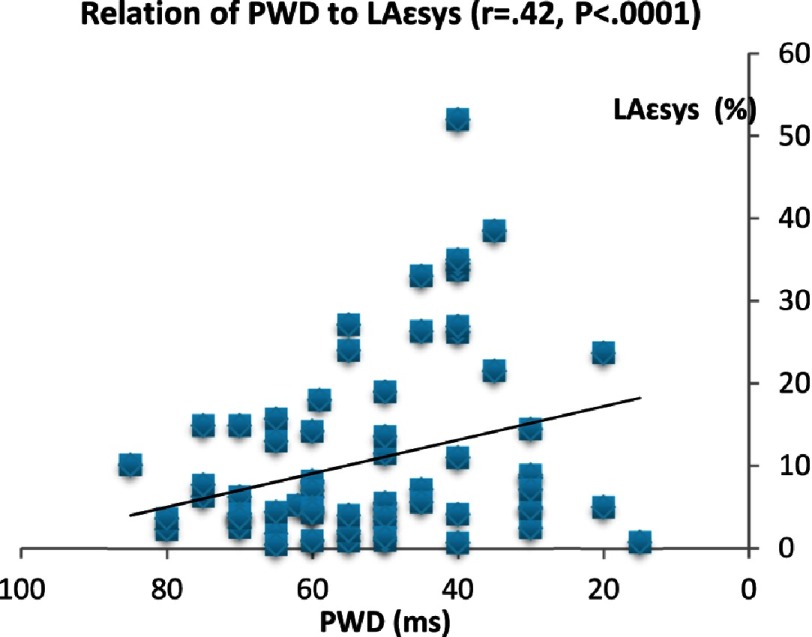
Relation of PWD to LAε_**sys**_.

**Figure 3F.**
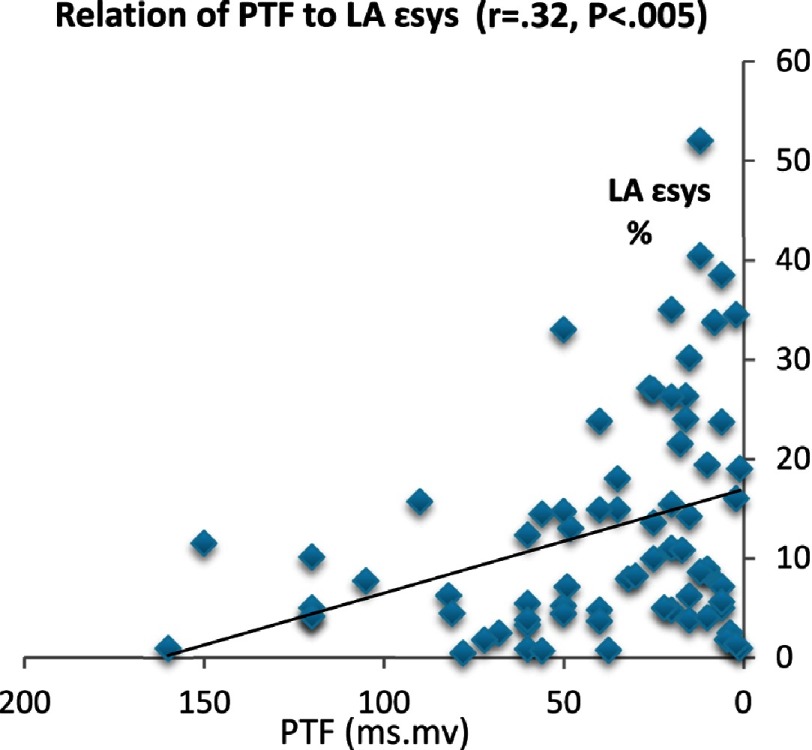
Relation of PTF to LAε_**sys**_.

**Figure 3G.**
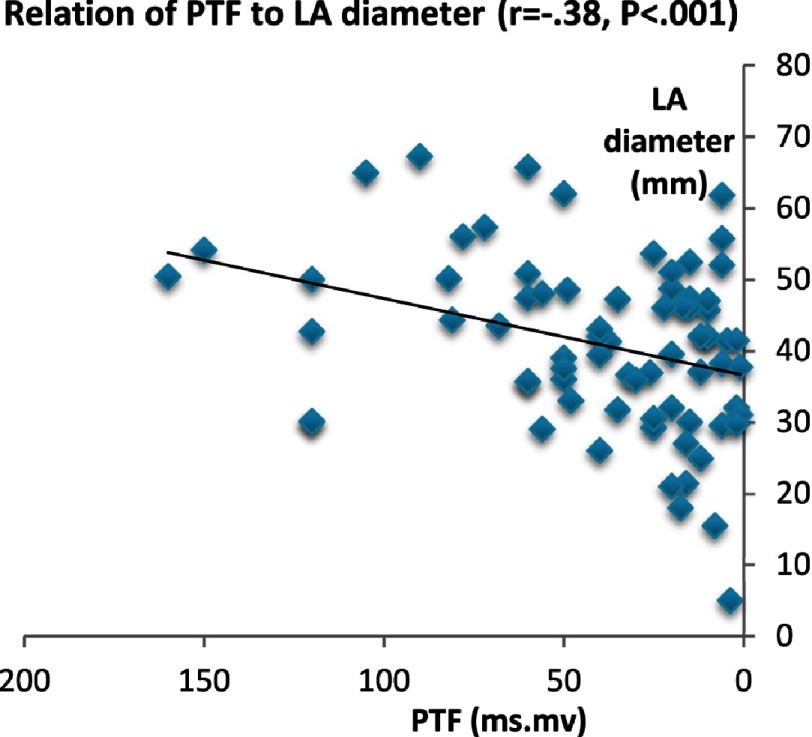
Relation of PTF to LA diameter.

### Multivariate analysis

Stepwise forward, multiple linear regression analyses were performed in IDCM, to find the main determinants of LA electromechanical delay. By this model, after adjusting potential determinants, LA reservoir function (LA ε_sys_) [*β* = 0.754; CI at 95%: 0.356–0.780, *P* < 0.001] and LA volume [*β* = 0.743; CI at 95%: 0.423–0.75, *P* < 0.001], and PWD [*β* = 0.848; CI at 95%: 0.311–0.644, *P* < 0.0001], and PTF [*β* = 0.927; CI at 95%: 0.357–0.722, *P* < 0.0001] are independent predictors for LA electromechanical delay.

### Receiver operating characteristic (ROC) curves and optimal cut off points for atrial electrical dispersion

**Figure 4.**
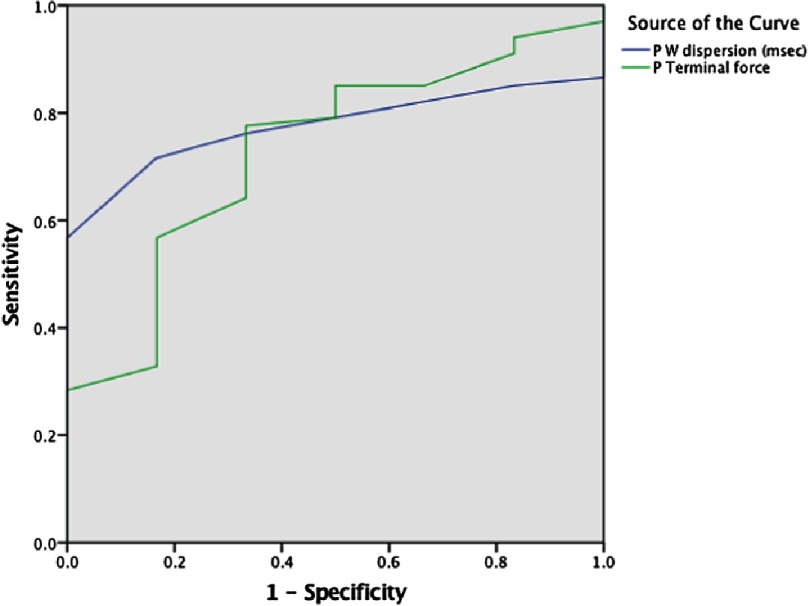
ROC curve for optimal cutoff value of PWD & PTF to discriminate electromechanical delay in IDCM.

To explore the cutoff points that discriminate IDCM patients with atrial electromechanical delay, we constructed ROC curves for PWD and PTF (Figure 8). PWD>54 ms shows 71% sensitivity and 75% specificity respectively. AUC: 0.776 [CI [0.669–0.883], *P* < 0.002], and PTF>45 ms shows 73% sensitivity and 74% specificity with AUC 0.730 [CI [0.540–0.920], *P* < 0.001] (Figure 4).

## Discussion

In this study, IDCM patients have longer P-wave duration, PR interval, higher PWD, PTF and atrial electromechanical delay (TTP-d), in addition to impaired atrial mechanics, than do healthy subjects. Besides, the PWD and PTF were predominantly correlated to LA volume and electromechanical delay. An interesting finding of this study is that LA size, LA reservoir function and PWD were the main predictors of atrial electromechanical delay in IDCM patients.

In this study, atrial conduction times in IDCM were examined by two non-invasive methods, electrocardiographic, using P-wave duration, PWD & PTF and the 2D strain imaging. All were found to be prolonged. To our knowledge, this study is the first to investigate atrial electromechanical conduction times, using 2D strain, in IDCM and its relation to P-wave atrial activity on surface ECG.

Prolonged intra- and inter-atrial EMD durations are known to be associated with inhomogeneous and discontinuous progression of the sinus impulses^9^. Prolonged atrial EMDs were reported in various diseases especially paroxysmal AF, mitral stenosis, and type I diabetes mellitus. This method is used as an early marker for detection of AF and elevated EMD is considered to be a risk factor for AF development^7,9,13^.

### LA functions in IDCM

IDCM is one of most common causes of heart failure and sudden death, it remains the leading indication for cardiac transplantation^14^. IDCM is a primary myocardial disease that predominantly causes dilatation and contractile dysfunction of the left or the right ventricle, or both without identifiable causes^14^.

Atrial function plays an important role in maintaining optimal ventricular function, and measurement of the LA diameter and volume by 2D echocardiography has been traditionally used for assessment of LA function. In the current study LA remodeling is the principle finding, as LA diameter and volume are markedly increased in comparison to control.

LA serves its role throughout the cardiac cycle by three functions, reservoir, conduit and contractile function. These LA functions have a great impact on LV diastolic function, LV filling and overall cardiovascular performance^15^.

In recent years, novel post-processing imaging methodologies have emerged, providing a complementary approach for the assessment of the LA. Atrial strain and strain rate obtained using 2D strain echocardiography have proved to be feasible and reproducible techniques to evaluate LA mechanics^15^. In the present study, the three components of atrial function showed marked deterioration in IDCM patients compared to healthy subjects.

This is in accordance of Limongelli et al^16^, they investigated twenty-four patients (mean age: 23 ± 16 years) with clinical diagnosis of non-ischemic dilated cardiomyopathy (DCM). In addition to ECG, conventional echocardiography, exercise stress test and Holter monitoring, LA strain and strain rate was measured. The authors demonstrated that LA strain and strain rate were significantly reduced (*P* < 0.01) and there is an inverse correlation between LA strain and PR interval (*P* < 0.01).

Gluer et al investigated^17^ LA deformation in 49 patients with non-ischemic cardiomyopathy using 2D strain imaging and measurements of NT-pro-BNP levels. The patients were divided into two groups consistent with LVFP, according to E/A ratio, E velocity, and E/E’ ratio. They concluded that 2D-STE-based LA function is impaired in patients with non-ischemic DCM. Additionally, LA reservoir and pump function parameters together with NT-pro-BNP levels might be useful in estimating LVFP.

### Atrial electromechanical delay in IDCM

LA mechanical dispersion was recently evaluated using atrial electromechanical delay (EMD) which is not only a reflection of atrial mechanics but point toward electrical inhomogenities and propensity to development of atrial fibrillation^17,18^. In the present study, EMD was investigated using the difference between LA segmental TTP and the standard deviation of its mean value (TTP-SD). These indices were markedly prolonged in comparison to control subjects. They were correlated inversely to LA reservoir, conduit function and contractile function.

There are few studies that have investigated the atrial conduction in patients with heart failure. Waggoner and coworkers^19^ demonstrated that PA lateral (from P-wave on ECG to peak atrial myocardial velocity) was delayed compared with the controls in patients with heart failure (ischemic or non-ischemic). Van Beeumen and coworkers^20,21^ demonstrated the prolongation of PA lateral, PA septal, and right atrium EMD in patients with DCM; however, they found that PA tricuspid and RA EMD were similar in the control and DCM groups. Furthermore, Pala et al reported that, atrial EMD using TDI was significantly prolonged in patients with non-ischemic DCM and it was positively correlated with the LA maximal volume, log B-type natriuretic peptide and negatively correlated with left ventricular ejection and E-wave deceleration time^22^.

NT proBNP is released from the cardiac ventricles in response to stretch and increase LV filling pressures^23^. This association can represent the elevated intra-atrial pressure and increased wall stress, which, in turn, may also affect atrial EMD.

### Relation of atrial EMD to P-wave dispersion

In IDCM, besides morphologic remodeling, myopathic process also causes electrical remodeling of the atria including depressed excitability, increased refractoriness, and conduction slowing or block^24–26^. Congestive heart failure is also associated with abnormalities of cell electrophysiology such as cellular uncoupling and anisotropy^27^. Atrial remodeling seems to be responsible for the conduction delay and atrial arrhythmias. In our study, we observed increased *P*_max_, PR interval, PWD, and PTF in patients with DCM. These electrocardiographic parameters have been associated with morphologic changes such as enlarged LA in addition to electrical remodeling^27,28^. Increased LA size causes a prolongation of the total depolarization time of the LA. Our findings including significant association between LA volume, PWD, and PTF support this conclusion.

P-wave dispersion is an ECG marker that reflects heterogeneous and slow atrial conduction by detecting abnormal atrial conduction with ECG leads of different orientation. Several studies demonstrated an increased P-wave duration in patients with heart failure, and marked PWD was found as a reliable predictive marker of atrial fibrillation^21^. In addition, Liu et al^28^ reported that PTF was significantly prolonged in patients with paroxysmal atrial fibrillation.

De Vos and coworkers^29^ reported that prolonged atrial EMD using TDI predicted the development of new-onset atrial fibrillation in their study, which comprised of 249 patients. In addition, prolonged atrial EMD in patients with paroxysmal atrial fibrillation was demonstrated with TDI, M-mode, and pulsed-wave Doppler echocardiographic studies^24–29^. Atrial EMD values were correlated with LA long axis in patients with paroxysmal atrial fibrillation.

These studies and our results suggest that prolonged atrial EMD is usually related to impaired atrial mechanics as a result of long lasting impairment of LV functions. This electromechanical prolongation can be reflected as electrical dispersion on surface ECG. Consequently, electromechanical dispersion as indicated by electromechanical delay by 2D strain imaging, and P-wave dispersion on surface ECG may be associated with the risk of developing atrial fibrillation in patients with IDCM.

In this study, we investigated the association between atrial EMD and the electrocardiographic parameters. Although EMD was moderately correlated with LA reservoir, conduit and contractile function, only PWD and PTF are independently associated with atrial EMD. It seems that increased atrial electrical dispersion reflects an electromechanical delay that results as a consequence of atrial dilation and dysfunction. On the other hand, the prolonged depolarization and impaired conduction, as represented by prolonged PR interval, could be another deleterious outcome of atrial dilation and dysfunction.

### Study limitations

Since there was no specialized software for LA strain analysis, the software for LV analysis was used. This might influence the echocardiographic results. For 2D-strain imaging study of the LA, obtaining optimal images was sometimes challenging especially in obese patients. Although conducted in a very specific patient group, another limitation was the small size of the study population. Moreover, even though echocardiographic estimation of LVFP, using E/E’ has been shown to be comparable with invasive methods in evaluating LVFP, measurements of pro BNP and its relation to electromechanical delay is lacking. Finally Long term follow up to estimate development of atrial fibrillation was important to confirm the relation to electrical dispersion and patient risk stratification.

## CONCLUSIONS

The left atrial mechanical function (which is well established to be independent prognostic factor) significantly decreased in patients with IDCM in comparison with normal subjects. Also, intra-atrial electromechanical delay gets longer in IDCM and is correlated with PWD and PTF. Atrial electromechanical delay is influenced by atrial remodeling and reflected as PWD on surface ECG in IDCM. P-wave indices, especially P-wave dispersion and P terminal force (PTF) can be used as accurate, simple and noninvasive method to predict the LA electro-mechanical activity in IDCM patients.
